# PACE4-altCT isoform of proprotein convertase PACE4 as tissue and plasmatic biomarker for prostate cancer

**DOI:** 10.1038/s41598-022-09778-6

**Published:** 2022-04-11

**Authors:** Frédéric Couture, Luojun Wang, Frédérik Dufour, Keena Chabot-Maheux, Nadia Ekindi Ndongo, Robert Sabbagh, Robert Day

**Affiliations:** 1 Department of Pharmaceutical Sciences, TransBIOTech, Lévis, QC G6V 6Z3 Canada; 2 Department of Pathology, Université de Sherbrooke, Sherbrooke, QC Canada; 3 Faculté de Médecine et des Sciences de la Santé, Université de Sherbrooke, Sherbrooke, QC J1H 5N4 Canada; 4 Division of Urology, Department of Surgery, Université de Sherbrooke, Sherbrooke, Canada; 5 Phenoswitch Bioscience Inc, Sherbrooke, QC J1G 5J6 Canada

**Keywords:** Prostate cancer, ELISA, Immunohistochemistry

## Abstract

The proprotein convertase PACE4 has demonstrated value as a viable therapeutic target in prostate cancer (PCa). A novel isoform named PACE4-altCT, which arises in neoplastic lesions, plays an important role in tumor progression and has been validated as a pharmacological target. With the discovery of its overexpression in PCa and the alternative splicing of its pre-RNA to generate an oncogenic C-terminally modified isoform named PACE4-altCT, understanding and validating its value as a potential biomarker is of great interest either from prognostic or targeted therapy intervention. Expression of ERG in LNCaP cells was used to investigate the relationship between ERG expression occurring in PCa cells and PACE4-altCT expression by Western blot and qPCR. Using immunohistochemistry, the expression levels of PACE4 isoforms in patient tissues were investigated and correlated with ERG tumor status and Gleason score. An ELISA method was developed using affinity purified recombinant protein and used for quantitative analysis of plasma concentrations of PACE4-altCT and used for correlation. In contrast with the consensual isoform, PACE4-altCT was only strongly overexpressed in prostate cancer patients, correlated with ERG expression levels. Despite its intracellular retention PACE4-altCT could be detected in the plasma of most patients with prostate cancer, whereas it was only found at low levels in normal patients whereas total plasmatic PACE4 levels did not vary significantly between groups. Our study demonstrates that PACE4-altCT is strongly overexpressed in prostate cancer using both immunohistochemical and ELISA techniques and may have some interesting potential as a biomarker.

## Introduction

Prostate cancer (PCa) remains an important proportion of the diagnosed cancers every years as it now accounts for 1 in 8 new diagnoses in men^1^. Despite the fact that since 1993, PCa-associated death rates dropped by more than 50%, it remains the third cause of cancer-associated death in men behind lungs cancers. These statistics strongly suggest that the identification of alternative (or complementary) biomarkers that could accurately distinguish between aggressive PCa and indolent forms is required to minimize overdiagnostic impact on patients. As of today, the most popular biochemical test for PCa is the prostate specific antigen (PSA) blood assay, which is sensitive but lacks specificity and correlation with aggressive forms of the disease^2,3^.

Recent works from our group have highlighted the marked implication of the proprotein convertase (PC) PACE4 in the progression of PCa tumors^4,5^. This serine protease is overexpressed by PCa cells and regulates cell-growth capabilities. More precisely, PACE4 overexpression carries the generation of an alternatively spliced isoform in cancer cells that harbors a distinctive protein C-terminal called PACE4-altCT^6^. This isoform is just as active as its consensual isoform but is intracellularly retained, which as a result makes it support PACE4-activity associated proliferation in the tumor cells. At the moment, the mechanism involved in PACE4 overexpression and stimulation of alternative splicing are not fully understood. The DNA methylation status in the surrounding of the alternative terminal exon stimulates the integration of this exon by splicing through CTCF binding^6^. Yet, the driver of PACE4 gene expression in prostate cancer are not yet identified^7^.

In the succession of events leading to prostate adenocarcinoma development, one of the two most important cellular events, along with the loss of phosphatase and tensin homolog (PTEN) gene, is the genomic rearrangement leading to the fusion between transmembrane protease serine 2 (*TMPRSS2*) with the v-ets erythroblastosis virus E26 oncogene homolog (*ERG*), also termed *TMPRSS2:ERG,* which occurs in 50–60% of PCa tumors^8–10^. This gene fusion results in the expression of the oncogenic ERG protein in PCa cells (Supplementary Fig. 2, normal prostate cells being negative), which enables carcinogenesis progression by promoting proliferation, angiogenesis and differentiation-related processes^8^. The presence of ERG in prostate tumor is known to be associated with significant changes in DNA methylation^11,12^ and has been associated as an unfavorable outcome in numerous studies^9,10^. Consistent with these observations and the known involvement of PACE4 in prostate adenocarcinoma progression, we aimed to evaluate the relationship between both ERG expression and PACE4-altCT expression in prostate primary tumor tissues and its potential as a biomarker using biochemical recurrence as an outcome.

Moreover, knowing the newly described isoform of PACE4 generated in PCa cells, we investigated whether PACE4, as well as the PACE4-altCT isoform, could serve as a biomarker for PCa either as a tissue or as a blood biomarker. We thus turned toward the development of an isoform specific ELISA to detect PACE4-altCT in the plasma from patients and used it to test the hypothesis that PACE4-altCT in preoperative plasma specimen could predict the outcome.

## Material and methods

### Tissue microarray construction (TMA) and immunohistochemical analysis

Formalin-fixed paraffin-embedded (FFPE) archived prostate specimens from 95 patients were used to build TMA (tissue microarray, see Supplementary methods) and were used for IHC (immunohistochemistry) analysis. The patients underwent radical prostatectomy between 2006 and 2011 at the Centre Hospitalier Universitaire de Sherbrooke. The research protocol, consent forms and all methods were approved by the Institutional Review Committee for the Use of Human Resected Material at the Centre Hospitalier Universitaire de Sherbrooke (approval #10-017 and #12-151). All methods were performed in accordance with the relevant guidelines and regulations. Patients that agreed to participate and in this study freely signed an informed consent form. FFPE tissues IHC images for PACE4 IHC staining were performed as described in Couture et al.^6^ and were analyzed by 3 independent observers, including expert pathologists, using a pre-established scale for staining evaluation. Each core was scored individually and the highest score from duplicate core of each specimen for each observer were used to calculate an averaged immunoscore. ERG scores were assessed on a simple single score scaling system (0 = negative, 1 = low, 2 = high).

### Production and purification of recombinant PACE4 constructs

For each purification, 100–150 mL of conditioned medium of Schneider 2 cells, stably expressing construct encoding hPACE4-FL and hPACE4-altCT cDNA C-terminally tagged with 6xHis-V5 in pAC5.1 vector, were buffer-exchanged and concentrated on 30 kDa molecular filtering centrifugal devices (Centricon Plus-70, Millipore Sigma) and purified on a nickel chelating-resin (see Supplementary methods). PACE4 concentration in recombinant protein preparations were determined by quantitative LC–MS/MS methods monitoring PACE4 tryptic peptides (see Supplementary methods).

### Plasma collection

Blood samples were obtained from patients who agreed to participate prior to the prostatectomy procedure. For control healthy patients, healthy control samples were collected from patients referred by a medical doctor for a PSA dosage who agreed to donate some blood and signed an informed consent form. Blood was collected in EDTA coated tubes (Vacutainer; BD) and centrifuged 15 min at 5000 × *g* (4 °C). Plasma was then aliquoted and stored at − 80 °C until use for ELISA assay (described in Supplementary methods).

### Cell culture and transfections

LNCaP PCa cells were obtained from the American Type Culture Collection (ATCC, Mannasas, USA) and cultured in RPMI 1640 with 10% fetal bovine serum (FBS; Wisent Bioproducts, St Bruno, QC). Transfections, RT-qPCR and western blot were performed as reported in Couture et al.^6^ (see precisions in Supplementary methods)^6^.

### Statistical analysis

Statistical analyses were performed using GraphPad Prism 7 software using unpaired. Student *t*-tests were used for means comparison. Pearson (for continuous variables, e.g. plasmatic concentrations) or Spearman (for ordinal variables, e.g. immunoscores, Gleason scores) correlation analyses were used to calculate *r* coefficients. For Kaplan–Meier, patient surgery date and the date at which the first PSA values > 0.2 ng/mL was observed were used for biochemical recurrence-free survival analysis. Log-rank (Mantel-Cox) test was used to determine the P-values. For all tests performed, *P* value < 0.05 were considered statistically significant.

## Results

### Impact of ERG expression on PACE4 alternative splicing in LNCaP cells

**Figure 1:**
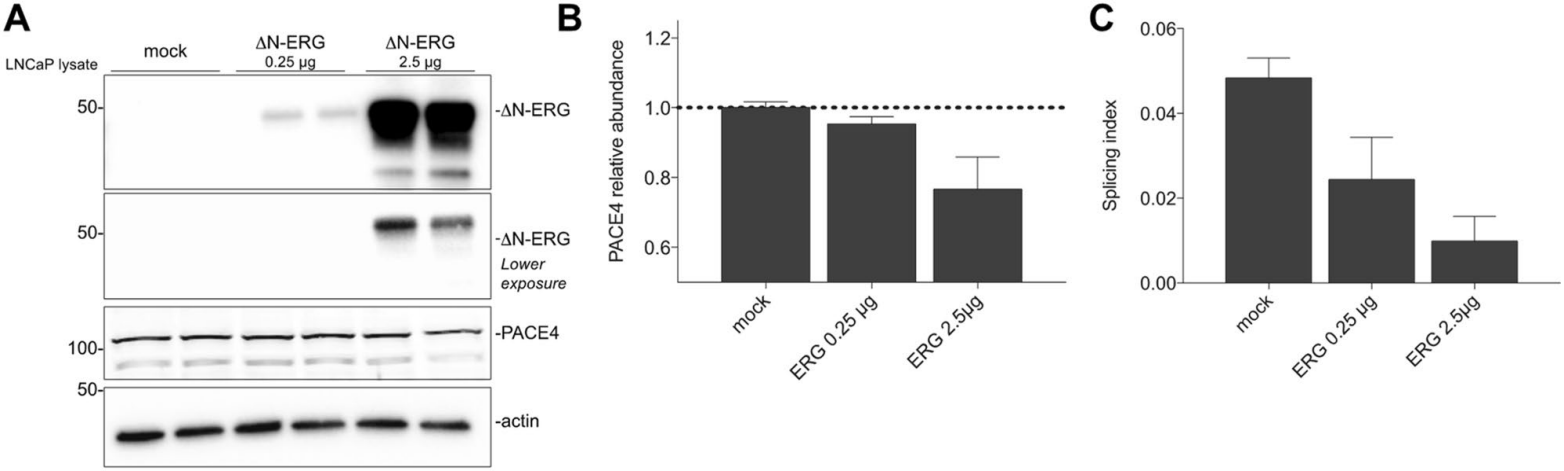
Relationship between PACE4-altCT and ERG status in prostate cancer cells. (**A**) Western blot analysis on LNCaP cell lysates following transfections with the indicated vectors. Each lane represent an individual transfection. ERG is shown with distinct exposures. These blots were cropped to emphasize the intended band. Uncropped original blots are provided as in Supplementary Material. (**B**) PACE4 densitometry analysis using actin as a reference. Data are mean ± SEM. (**C**) PACE4 splicing indices determined by RT-qPCR in transfected LNCaP cells. Data are mean ± SEM (n = 3).

To investigate the relationship between ERG expression and PACE4 alternative splicing, LNCaP cells, which are negative for ERG and TMPRSS2/ERG fusion, were transfected with either 0.25 µg or 2.5 µg of plasmid encoding the cDNA of the TMPRSS2/ERG fusion product: ΔN-ERG. After transfection, Western blot analyses revealed that cell lysates blotted for PACE4 showed a ERG-dependent dose–response reduction in terms of intracellular PACE4 (Fig. 1A,B), which in these cells is known to be mostly composed of PACE4-altCT^6^. mRNA analyses by RT-qPCR were also supportive of these observations with dose–response reduction in the splicing indexes (ratio between PACE4-altCT/PACE4-FL mRNA levels; Fig. 1C). This therefore suggest that PACE4-altCT genesis may be negatively impacted with ERG expression taking place during prostate carcinogenesis.

### Immunohistochemical analyzes of PACE4 isoforms in a PCa patient cohort tissue microarray

To investigate this possibility in a clinically representative context, formalin-fixed paraffin-embedded (FFPE) prostate tissues from 95 patients (who had a prostatectomy between 2006 and 2011) were retrieved and used to build a tissue microarray (TMA) containing duplicate cores from each tumor (with cores from both primary and secondary Gleason grades, when applicable), prostate intraepithelial neoplasia (PIN) or normal tissues.

**Figure 2:**
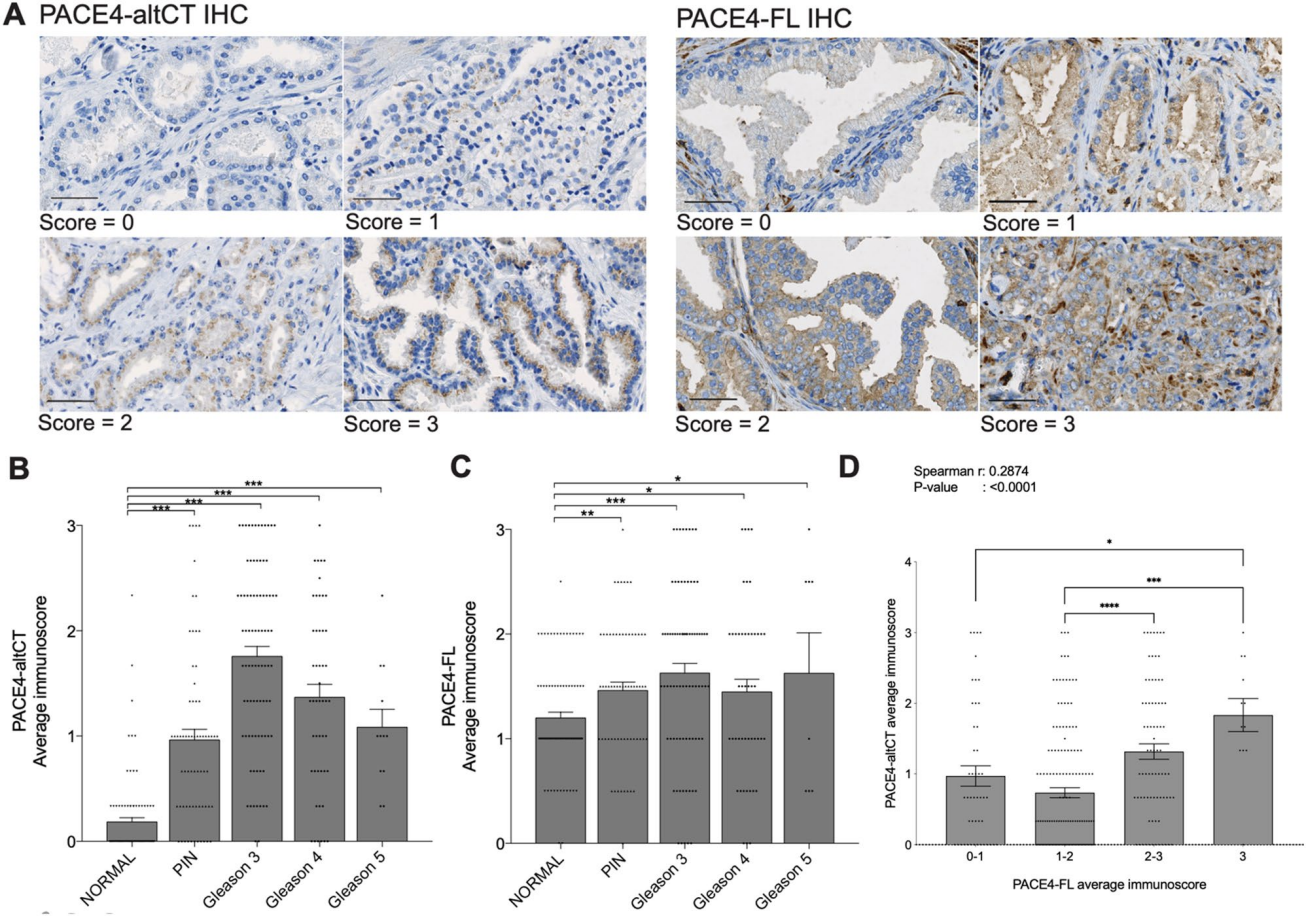
PACE4 isoforms immunohistochemical analysis of PCa specimens. (**A**) Representative images of prostate epithelial cells stained for either PACE4-altCT or PACE4-FL with the different scoring units used. Magnification: 20 ×, scale bars represent 50 µm. A 40 × magnification figure is also found in Supplementary Fig. 1 to encompass cellular localization of immunohistochemical signal. (**B**) Averaged immunoscores for PACE4-altCT on the TMA cores of normal (n = 92), PIN (n = 67), Gleason 3 (n = 85), 4 (n = 49) and 5 (n = 12). Gleason scores are the primary Gleason pattern for each patients. Data are means ± SEM, all individual data are shown as dots. (**C**) Averaged immunoscores for PACE4-FL on the TMA cores of normal (n = 88), PIN (n = 64), Gleason 3 (n = 79), 4 (n = 47) and 5 (n = 8). Differences between the number of patients analyzed for this figure compared to PACE4-altCT (Fig. 2B) are due to core detachment and/or histological differences on successive slides. (**D**) Plots of immunoscoring for PACE4-altCT and PACE4-FL immunoscores for each tumor scored. The Spearman correlation r is calculated from the entire data set without regrouping scores together in intervals, histogram are means ± SEM. Statistical analysis is based on One-way ANOVA.

PACE4 isoforms levels were first investigated using previously validated^6^ antibodies directed against either PACE4-FL or PACE4-altCT using TMA sections. The epithelium from each core was scored using a 0–3 grading system (Fig. 2A) to yield Fig. 2B,C for each PACE4 isoforms. PACE4-FL, which is expressed by most tissues, stained positively (1–3) in > 92% of the analyzed cores whereas PACE4-altCT positivity was clearly and statistically much stronger in tumor cores than in the normal prostate cores. Interestingly, statistically significant elevation in immunoscoring could be depicted between PIN, Gleason 3, 4 and 5 foci compared to normal (Fig. 2C). IHC showed that 96% (91/94 with available data) were positive in either primary or secondary tumor pattern, with 94% (89/94) when only looking at primary patterns This is coherent with the previously reported increase in PACE4 expression levels in PCa, either at the mRNA levels or at the protein levels by IHC^13,14^. In Kang et al.^14^, no difference could be observed between PACE4 staining (measuring total PACE4) in normal glands and PIN. This difference may be due to the fact that in the present study the different stages (normal, PIN and cancer) are derived from the same patient specimen, thus reducing heterogeneity bias.

When stained for PACE4-altCT, mean immunoscores were much higher in the Gleason 3–4–5 PCa and in PIN than those observed in normal glands (which were mostly negative (Fig. 2B). The highest scores were observed in Gleason score 3 PCa foci. The difference between normal glands and all conditions tested was much higher than the one observed for PACE4-FL. Interestingly, levels of each isoforms correlated one with the other when comparing all tissues tested (Spearman r: 0.2874, *P* value < 0.0001) as well as only the tumoral tissues (Spearman r: 0.2047, *P* value: 0.0190; Table 1), further emphasizing the tight isoform-based relationship between these two proteins (Fig. 2D). Neither PACE4-altCT nor PACE4-FL immunoscores correlated with PSA levels at diagnosis (Table 1), margin status and tumor TNM.

**Table 1:** Correlation analysis between tissues immunoscores.

Correlation	With	Spearman	
		r	*P* value
**IHC correlation analysis (all tissue types)**			
PACE4-altCT	PACE4-FL	0.2874	< 0.0001
	ERG	0.4433	< 0.0001
PACE4-FL	ERG	0.1724	0.0101
**IHC correlation analysis (tumors only)**			
PACE4-altCT	PACE4-FL	0.2047	0.0190
	ERG	0.3048	0.0002
PACE4-FL	ERG	0.0781	0.3753

### PACE4-altCT correlates with ERG expression in PCa

**Figure 3:**
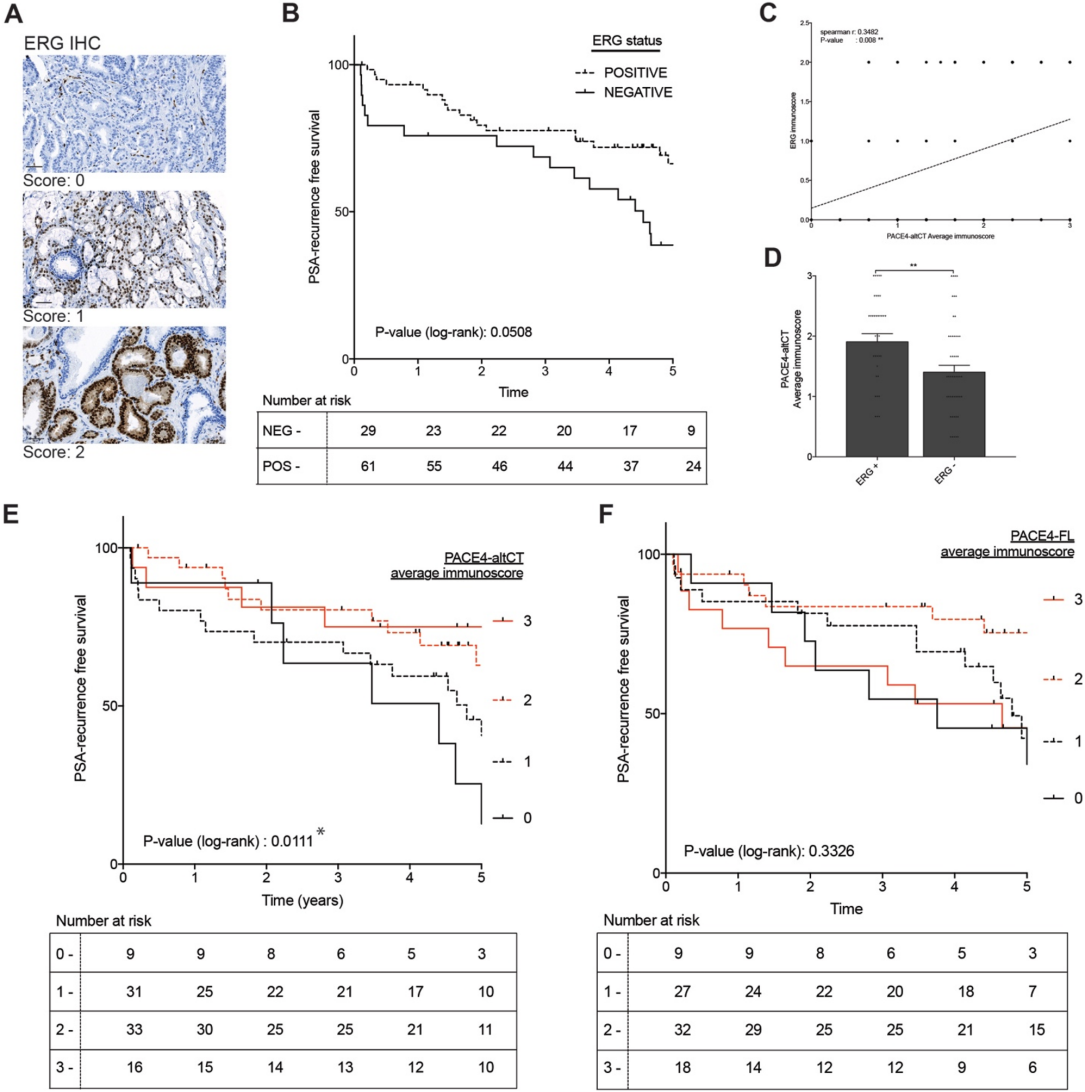
PACE4-altCT relationship with tumor ERG status and biochemical recurrence in prostate adenocarcinomas. (**A**) Representative images of prostate epithelial cells stained for ERG with the different scoring units used. Magnification: 20x, scales represent 50 µm. (**B**) Kaplan–Meier analysis of patient biochemical recurrence (PSA-based) according to their tumor primary Gleason pattern immunoscores for ERG. Gehan-Breslow-Wilcoxon test P-value: 0.0282. (**C**) Correlation analysis between PACE4-altCT and ERG immunoscores in the analyzed cores. All individual coordinates are shown (but are overlaid for identical data). The dashed line represent the linear regression. (**D**) Averaged immunoscores of tumor cores according to their ERG status. ERG + includes both ERG immunoscores 1 and 2. (**E**) Kaplan–Meier analysis of patient biochemical recurrence (PSA-based) according to their tumor primary Gleason pattern immunoscores for PACE4-altCT or PACE4-FL (**F**). Log (rank) Mantel: * indicates *P* value < 0.05, ** < 0.01 and *** < 0.001.

We next investigated the occurrence of ERG expression by IHC on our TMA, which is demonstrative of the TMPRSS2/ERG gene fusion and Supplementary Fig. 2^15^. ERG expression was scored for high and low staining intensity (Fig. 3A). In our cohort, 67% (61/90 patients) of the cases were positive for ERG expression, which is consistent with the usual incidence rate of this gene fusion in PCa patients^8–10^.

Using immunoscoring, correlation analyses were performed and demonstrated that ERG levels positively correlate with PACE4-altCT in all tissues types (Spearman r: 0.443, *P* value: < 0.0001) and when solely using tumoral tissues (Spearman r: 0.3048, *P* value: 0.0002; Fig. 3C,D). ERG levels also correlated with PACE4-FL but to a lower extent when looking at all tissues (Spearman r: 0.1724, *P* value: 0.0101).

Despite the fact that both TMPRSS2/ERG fusion or ERG expression status are reported as bad prognostic when investigated using incidence of metastasis or patient death^15,16^, they have been described as indicators of good prognosis when using biochemical recurrence when evaluated with regards to biochemical recurrence^17,18^. In our cohort, similar results were obtained (Fig. 3B) showing a very clear trend (*P* value: 0.0508) toward enhanced time prior to biochemical recurrence for ERG-positive tumors. When assessed for patient biochemical-free survival time according to PACE4-altCT immunoscores, a statistically significant inverse association (Log-rank for trend *P* value: 0.0111) was depicted suggesting that patient negative or having low tumor PACE4-altCT levels were more susceptible to biochemical recurrence than those with higher levels (Fig. 3E). No significant association could be established with tumor PACE4-FL levels (Fig. 3F). These results suggest that in prostate adenocarcinoma ERG expression co-occurs with PACE4-altCT despite the apparent lack of causality between the two events knowing that ERG expression tends to slightly reduce PACE4-altCT levels in cells (Fig. 1).

### Plasma PACE4 isoforms levels detection by sandwich ELISA

PACE4-altCT being overexpressed in PCa tumors (Fig. 2B and previous work)^6^, we sought to investigate its presence in plasma from patients through the use of an ELISA assay to detect both total PACE4 levels as well as PACE4-altCT levels. One study previously reported that PACE4 could be detected in the serum of some PCa patients using an IP-MS/MS approach. However no such attempt or demonstration has ever been reported for PACE4-altCT^13^. Despite the fact that PACE4-altCT isoform is known to be intracellularly retained in cancer cells, many biomarkers are actually found in the circulation after tissue structure disorganization and/or cell necrosis (e.g. CA125 or S100B)^19,20^.

To design and experimentally validate such an ELISA approach, both recombinant isoforms were first isolated from Scheider 2 (S2) conditioned media stably expressing as 6 × His tagged protein using nickel chelating resin (See Supplementary methods). To determine the exact concentration of PACE4 in each purified recombinant isoform protein batches, a quantitative mass spectrometry (MS) approach was developed, based two distinct tryptic peptides coming from the middle of the protein sequence (not specific to either isoform, see Supplementary Fig. 3). For each peptide (obtained by chemical synthesis; MLELSAPELEPPK and NVVVTILDDGIER), two standards curves were generated using two distinct MS/MS transitions (see Supplementary Methods) with R^2^ > 0.985. These standard curves were used to quantify the amount (in ng) of PACE4 in the different recombinant protein preparations that would serve as standards for ELISA assays.

**Figure 4:**
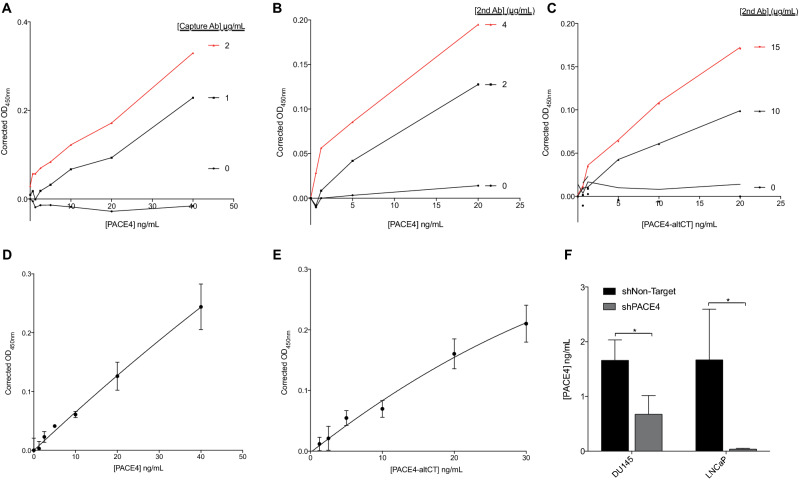
Development, optimization and validation of PACE4 sandwich ELISA assays. Antibody validation assays for (**A**) PACE4 capture antibody, (**B**) total-PACE4 sandwich (2nd) antibody and (**C**) PACE4-altCT sandwich (2nd) antibody. (**D**) Representative standard curves of total PACE4 and (**E**) PACE4-altCT. Assays from A-E use recombinant PACE4 (diluted in human serum diluted 1;5 with PBS). (**F**) Quantitation of PACE4 concentration in the conditioned culture media of control (shNon-Target) and PACE4-knockdown (shPACE4) DU145 and LNCaP PCa cells (n = 3). * indicates *P* value < 0.05. Data are means ± SEM.

Using a recombinant monoclonal antibody directed toward PACE4 catalytic domain as an ELISA capture antibody, assay conditions were set up and optimized to quantitatively measure total PACE4 (using a biotinylated anti-P-domain sandwich antibody) and PACE4-altCT (using a biotinylated anti-altCT sandwich antibody) concentrations in plasma. The PACE4-FL specific antibody did not yield satisfactory levels of sensitivity when combined with the monoclonal capture antibody to be useable in an ELISA assay (despite being functional and specific in IHC assay). Capture antibody (Fig. 4A) as well as both sandwich antibodies (Fig. 4B,C) showed PACE4-dependent variations upon dilutions and generated linear standard curves with R^2^ > 0.95 (Fig. 4D,E). Detection limits were of 0.5–1 ng/mL for both assay when done is plasma (established concentrations below the assay detection limit were valued as 0), but when performed in PBS or culture medium, the limits were of ~ 0.1 ng/mL. To further validate the assay, concentrations of total PACE4 were determined in conditioned media from control and PACE4-knockdowned DU145 and LNCaP PCa cells (Fig. 4F). PACE4 concentrations were reduced by more than 50% in medium conditioned by PACE4-knockdown cells (even undetectable in LNCaP shPACE4), confirming the assay specificity.

### Plasma PACE4-altCT levels correlate with tumor Gleason scores

**Figure 5:**
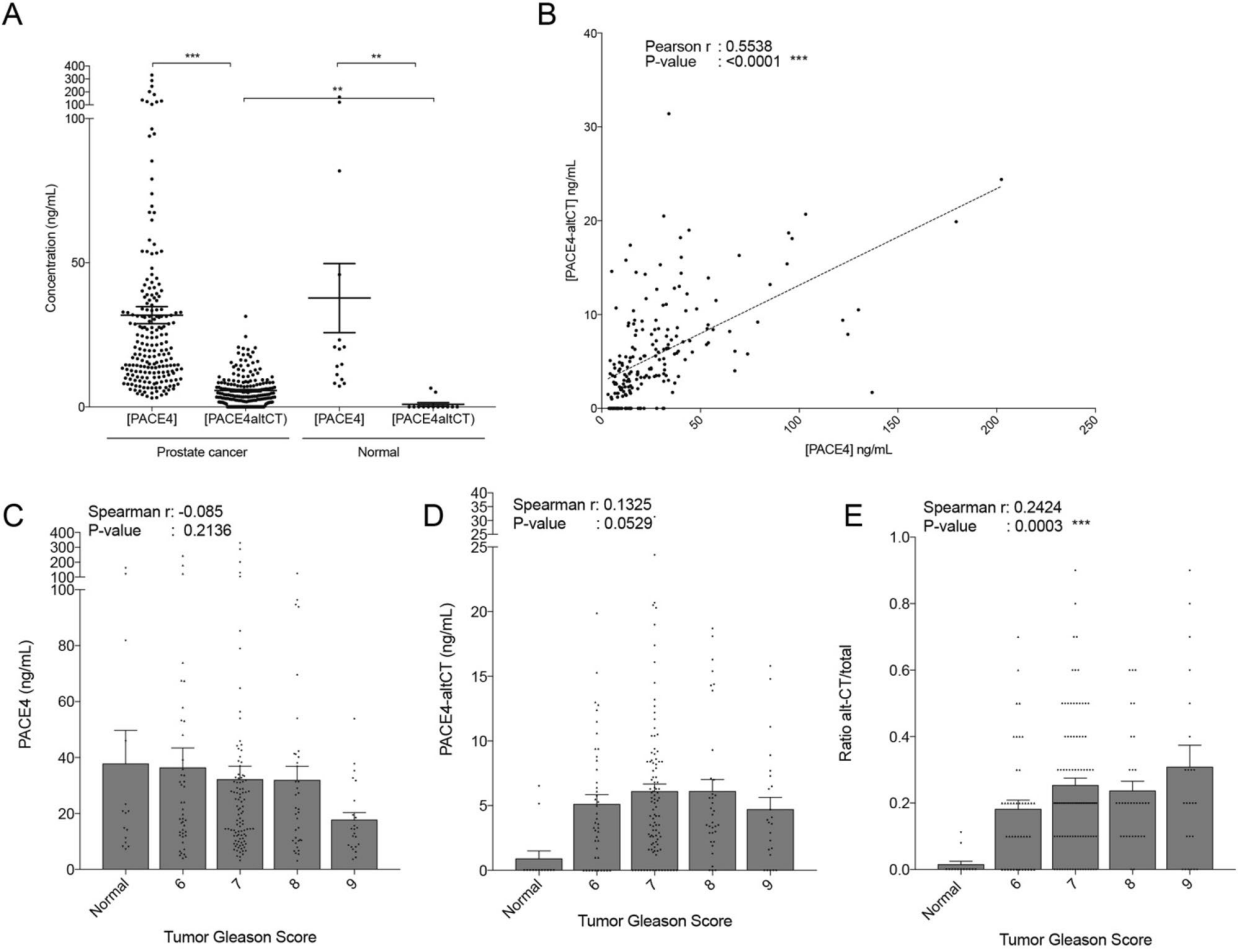
PACE4 and PACE4-altCT plasmatic concentration in PCa patients. (**A**) ELISA-determined concentration of total PACE4 and PACE4-altCT in plasma from PCa patients (n = 205) and normal patients (n = 13). Each individual data is shown as a dot, bars represent mean with SEM. (**B**) Correlation analysis between the concentration of PACE4 and PACE4-altCT for each PCa patient. The dashed line represents the linear regression. (**C**) Concentrations and correlation analyzes of total PACE4, (**D**) PACE4-altCT concentrations and (**E**) PACE4-altCT/total PACE4 ratios in the plasma of normal and PCa patients according to their respective tumor Gleason score. All data points are shown as a dots, normal n = 13, Gleason 6 (n = 43), Gleason 7 (n = 100), Gleason 8 (n = 33), Gleason 9 (n = 9). Bars are means ± SEM.

Levels of PACE4 and PACE4-altCT were further measured in plasma specimens collected from patients just prior to radical prostatectomy. Measured plasma levels of total PACE4 were much higher than PACE4-altCT levels (with averages of 31 ng/mL and 5.4 ng/mL respectively; Fig. 5A). Total PACE4 levels in plasma from normal and PCa patients were not different (averages of 31 vs. 37 ng/mL respectively), whereas PACE4-altCT levels were much more elevated in PCa when compared to normal patients (averages of 5.4 vs. 0.9 ng/mL respectively). Correlation analyses indicated that the levels of both isoforms correlated one with each other (Pearson r: 0.5538, *P* value: < 0.0001; see Fig. 5B and Table 2), but not with PSA levels. Total PACE4 plasmatic concentrations did not correlate with tumor Gleason scores (Fig. 5C). However, PACE4-altCT levels displayed a clear tendency to correlate with tumor aggressiveness (Spearman r: 0.1325, *P* value: 0.0529; Fig. 5D). Upon normalizing the portion of PACE4-altCT over the total circulating PACE4 (as a ratio PACE4-altCT/PACE4), a stronger correlation with tumor Gleason score could be depicted (Spearman r: 0.2424, *P* value: 0.0003; Fig. 5E) suggesting that reporting these values as ratios normalize the data together.

**Table 2:** Correlation analysis between plasmatic concentrations.

Correlation	With	Spearman	
		r	*P* value
**Plasma concentrations correlation analysis**			
PACE4-altCT	PACE4	0.4833	< 0.0001
	PACE4-altCT/PACE4 (ratio)	0.5042	< 0.0001
	Serum PSA	− 0.1255	0.0833
PACE4	PACE4-altCT/PACE4 (ratio)	− 0.1855	0.009
	Serum PSA	− 0.08919	0.2069
PACE4-altCT /PACE4 (ratio)	Serum PSA	0.01934	0.7853

## Discussion

PACE4 overexpression in PCa is required for sustained tumor growth^6^. Following alternative splicing driven by the methylation status of the intra-exonic region encoding the alternative C-terminal on the PCSK6 gene, the PACE4-altCT isoform is generated and supports cell proliferation. In this paper, we explored the question of whether these PACE4 isoforms could serve as potential biomarkers for PCa.

In non-small cell lung cancer (NSCLC), PACE4 expression in tissues was previously reported to carry a prognostic value regarding patient survival, patient with high PACE4 being having worsened prognostic^21^. It was thus justified to evaluate whether PACE4 could be a marker with some diagnostic or prognostic value in PCa as well. In 2012, it was reported that PACE4 could be detected in the serum of some PCa patients through stable isotope standards with capture by anti-peptide antibodies (SISCAPA) to detect tryptic peptide in multiplex immunoprecipitation (IP)-MS/MS assay^13^. Although this publication did not provide absolute measurements and could detect increased serum PACE4 levels in only up to 8 patients out of the 50 measured, it provided a proof-of-concept that PACE4 could be detectable in circulation and may display some potential as a blood marker. Also, we observed that PACE4-altCT is not secreted efficiently by cancer cells, which initially oriented us toward its use as a tissue biomarker. However, many useful serum biomarker proteins are not secreted but are still detectable in the circulation (e.g., HSP70, S100B, CA125)^19,20,22^. These proteins, in fact, get released by tumor cells upon either tissue structure disorganization, tumor cell necrosis or apoptosis, which often occurs as hypoxic conditions arise in solid tumors^23^.

This justified our interest in further exploring the measurement of PACE4-altCT in blood-derived specimens. Through the use of IHC targeting each individual isoform^6^, it was possible to show the increased protein expression of PACE4-altCT in PCa tumors when compared to benign tissues (Fig. 2A,B). This overrepresentation of the altCT isoform compared to the more discreet increase of the PACE4-FL isoform clearly suggest the enhanced splicing of PACE4 mRNA in PCa and is in agreement with data obtained at the mRNA levels^6^. Moreover, as corroborated by the tissue co-expression analysis, the splicing intensification seems to be accompanied by increased PACE4 gene transcription (Fig. 2D, Table 1).

As mentioned previously, the TMPRSS2/ERG chromosomal rearrangement on chromosome 21q22 causes to the displacement of the ETS family transcription factor downstream of the androgen-sensitive promoter of the TMPRSS2 gene leading to the overexpression of a N-terminally truncated ERG protein (ΔN-ERG). It is suggested that this genomic rearrangement plays a role in the transformation of the prostatic epithelium^24^. ERG expression is very common in PCa tumors and is a well characterized marker that bears paradoxal prognostic value when looking either at biochemical recurrence or at metastatic progression/cancer-related mortality^25^.

For this reason, we examined the relation between the expression of ERG and PACE4-altCT. In our patient cohort, ERG expression and PACE4-altCT staining correlated together (Fig. 3C,D, Table 1). A priori, this observation may appear conflictual with the reduction of PACE4 splicing observed following ERG overexpression in PCa cells (Fig. 1B,C) however, considering the very high level of exogenous ERG expression required for the slight (roughly 20%) reduction, this suggest the lack of direct causality between PACE4-altCT genesis and ERG expression in PCa tumors. This model also fails to mimic the several events leading to the genomic instability and epigenetic changes taking place in the genesis of prostate cancer where ERG is expected to play a role. Conversely, it has been reported that ERG expression occurs in PCa tumors having a distinctive DNA hypomethylation pattern, especially those characterized by increased histone deacetylases expression^26,27^. This latter suggests that ERG and PACE4 splicing are instead sharing common triggers, possibly related to the cancer cell DNA methylation status.

Considering the known oncogenic functions of PACE4-altCT in PCa cells, the actual observation that tumors found to be negative for this isoform at the protein level display the worst prognostic based on biochemical recurrence is hard to reconciliate (Fig. 3C,D). However, it appear that such inconsistency is similar to the one observed with TMPRSS2/ERG fusion event, which is reported as a bad prognostic when investigated using incidence of metastasis or patient death^15,16^. Interestingly, studies investigating the prognostic value of ERG status using biochemical recurrence showed that TMPRSS2/ERG fusion or ERG expression was instead indicative of good prognosis^17,18^, a phenomenon consistent the current observation in this cohort (Fig. 3B). These observations suggest that the use of biochemical recurrence as an endpoint might actually be problematic, notably knowing that only 8–40% of patient having biochemical recurrence are further facing prostate-cancer associated deaths^28–30^.

Since no patient from our actual cohort faced cancer-associated death, as this cohort of patients recruited at surgery is composed of patient mostly exhibiting low grade, and thus prone to surgical excision, PCa tumors, it is impossible to test this hypothesis with our current dataset. Therefore, it would be interesting to investigate in future studies the prognostic value of PACE4-altCT for patient death or incidence of metastasis in PCa to better define the role of PACE4-altCT as late-disease marker.

The development and validation of our PACE4-altCT specific ELISA permitted the confirmation that PACE4-altCT is not only increased in PCa tissues but that it can also be found in the bloodstream. This finding is of great interest on the diagnostic point of view since the levels of PACE4-altCT are almost undetectable in normal patients compared to total PACE4, which levels remain similar (Fig. 5). In our study, our total PACE4 levels also suggest that only a fraction of patients have increased PACE4 (Fig. 5A). However, PACE4-altCT displays much higher differences between normal and PCa patients. Compared to the SISPACA method used by Klee et al.^13^ and the IP-MS method used to titrate the recombinant protein preparations, our ELISA method offered enhanced sensitivity due to streptavidin-based detection on poly-biotinylated antibodies. Taken together, these two technologies (PACE4-altCT ELISA and IHC) will permit further investigation regarding the potential of PACE4-altCT as a biomarker on the prognostic point of view using different endpoint such as metastatic dissemination of PCa-associated death.

## Supplementary Information

Supplementary Information.
