# Supplementary material for: PACE4-altCT isoform of proprotein convertase PACE4 as tissue and plasmatic biomarker for prostate cancer
Source: Sci Rep. 2022 Apr 11;12:6066. doi: 10.1038/s41598-022-09778-6 (PMC9001653; doi:10.1038/s41598-022-09778-6)
Supplement: Supplementary file 1 — Supplementary Information. [file 41598_2022_9778_MOESM1_ESM.pdf]

Supplementary material

PACE4-altCT Isoform of Proprotein Convertase PACE4 as tissue and plasmatic biomarker for prostate cancer

Detailed Material & Methods

Tissue microarray construction and staining

For each prostate tissue, duplicate cores (1.5 mm diameter) of both tumor primary pattern and secondary pattern (when applicable) as well as PIN and normal prostate glands. H&E confirmed the nature of each cores following examination by an expert uropathologist. PACE4 antibodies were diluted 1/200, and ERG antibody (EP111; Dako Agilent Pathology Solutions) was use following manufacturer instructions. Stained sections were scanned with a Nanozoomer (Hamamatsu, Hertfordshire, UK) using Nanozoomer Digital Pathology software at a 40x magnification setting.

Detailed production and purification of recombinant PACE4 constructs

For each purification, 100-150 mL of conditioned medium of Schneider 2 cells, stably expressing construct encoding hPACE4-FL and hPACE4-altCT cDNA C-terminally tagged with 6xHis-V5 in pAC5.1 vector[1], were buffer-exchanged and concentrated on 30 kDa molecular filtering centrifugal device (Centricon Plus-70, Millipore Sigma) until >80% of the buffer composition was 50 mM HEPES, 500 mM NaCl, 5 mM β-mercaptoethanol, 8 M urea, 7 mM imidazole (pH 8.0). Proteins were incubated O/N with 5-8 mL of pre-washed nickel chelating-resin (ProBond, ThermoFisher) at room temperature (RT). Resin was then pellet by centrifugation 1,000 x g (5 minutes) and washed 4 times with wash buffer (50 mM HEPES, 500 mM NaCl, 5 mM β-mercaptoethanol, 8 M urea, 7 mM imidazole pH 5.9) and stacked on a gravity-flow column. An imidazole gradient (from 20 to 500 mM, in wash buffer) was manually applied as 10 steps of 3 mL each while fractions were collected by a drop collector. All fractions were screened by dot-blot for V5 immunoreactivity (Invitrogen, 1/10,000, O/N)

and positive fraction were pooled on a 30 kDa molecular filtering centrifugal device. Protein integrity and purity was analyzed by SDS-PAGE and Coomassie blue staining.

Titration of recombinant protein by quantitative mass spectrometry analysis

To determine exact PACE4 concentration in recombinant protein preparations, quantitative LC-MS/MS methods monitoring PACE4 tryptic peptides were used. Various volumes of protein preparations (5-25 µL) were diluted in 150µL 50 mM Tris buffer (pH 8.0) and sequentially treated with 10 mM DTT (30 min, RT), 15 mM iodoacetamide (30 min, RT shed of light) and again 10 mM DTT (30 min, RT) and digested O/N at 37˚C with trypsin/LysC (Promega) in a thermo-shaker. In parallel, a standard curve containing synthetic tryptic peptides: MLELSAPELEPPK and NVVVTILDDGIER, at concentrations ranging from 0.1 to 10 nM in 150 µL Tris buffer pH 8.0. Prior to injection, samples were cleaned by solid-phase extraction (Strata-X 33u polymeric reversed phase, 30mg/1mL) using the following procedure on a vacuum manifold (each solution was allowed to completely drain before adding the next one) : 1 mL ACN, 1 mL H2O 0.1% formic acid, acidified peptide solution, 1 mL H2O 0.1% formic acid, 50% ACN 0.1% formic acid: for elution). Peptide were further dried in a Speed-Vac system and reconstituted in H2O 0.2% formic acid, 3% DMSO and submitted to LC-MS/MS analysis on a QTRAP 6500 LC-MS/MS system (SCIEX) upon injection of 7µL on a 5 µL injection loop with a 60 min gradient of ethanol (0.1% TFA, 3% DMSO) . Multiple-reaction monitoring (MRM) methods selectively measuring two transition (Q1 and Q3) for each peptide; MLELSAPELEPPK (727.38-809.5 and 727.38-880.47) and NVVVTILDDGIER (721.90 – 313.19 and 721.90- 817,41) were used to determined area under the curve (AUC) used to draw standard curves and further determine protein concentration in sample digest. These MS-determined concentrations are average from the four determined concentrations (over four standard curve for each transition monitored) and were further used to calibrate the ELISA assay.

Antibody biotinylation

Antibodies were raised in rabbits against the PACE4 P-domain and the C-terminal domain of PACE4-altCT isoform using antigenic peptides (Pacific Immunocorp). Whole

bleed were affinity purified using immobilized antigenic peptide column and stored at -80˚C for long term storage. For biotinylation, antibodies were thawed and centrifuged at 17,500 x g for 5 minutes to remove any precipitate and buffer exchanged for PBS 300 mM NaCl pH 8.1 (>80% exchange) on a Amicon molecular filtering centrifugal device with a 30 kDa cut-off. For each mg of antibody, 140 µg biotin-amido-caproate N-hydroxysuccinimide (NHS) ester dissolved in pure molecular sieved DMSO (10 µg/µL) were added in the sample (still in the molecular filtering unit) and incubated 1h at 4˚C. Unreacted ester was quenched by the addition of 1/10 volume of 1 M Tris buffer (pH 7.4). Biotinylated sample was then buffer exchanged 3 times to PBS buffer (>80% exchange). Antibody concentrations were determined by bicinchoninic acid assay.

ELISA assay

Flat-bottom sorbent-coated (Nunc MaxiSorp high protein-binding, ThermoFisher) were first coated with 100µL (2µg/mL) anti-PACE4 catalytic domain recombinant monoclonal antibody (Abcam, ab151562) in 500 mM ammonium bicarbonate buffer (pH 9.5) O/N 4˚C with a plastic seal. Plates were then washed twice with bi-distilled water and blocked by incubating the plates 1h at RT with 200 µL of 5% skim milk in PBS (for total PACE4 ELISA) or 5% bovine serum albumin (BSA) in PBS (for PACE4-altCT ELISA). Plates were again washed with bi-distilled water and further used to apply 200µL of diluted sample. Samples were minimally diluted ½ (100µL sample + 100 µL buffer) with RIPA buffer (50 mM Tris, 150 mM NaCl, 0.1% SDS, 0.5% Na-Deoxycholate, 1% NP-40, 1% Triton X-100, pH 7.4 and 1X protease inhibitor; Roche Complete Mini). For standard curve, recombinant protein preparation were used to prepare a standard curve in normal human serum (Sigma) diluted ½ in RIPA buffer from which the PACE4 concentration (determined by ELISA using serial dilution with recombinant protein) was taken into account for the final concentration used in the calculation. For total PACE4 assay, purified PACE4-FL was used whereas for PACE4-altCT assay, purified PACE4-altCT was used for dilutions. Samples were incubated O/N 4˚C in the sealed plates. For conditioned media, 200 µL of sample were putted in each well (without RIPA buffer) to maximize detection. Plates were further washed 4 times with PBST (0.1% Tween-20) prior to the addition of rabbit biotinylated secondary antibody (see Detailed method). For

total PACE4 ELISA, 100µL of 4 µg/mL of biotinylated rabbit anti-PACE4 P-domain antibody in PBST containing 1% BSA and 0.4% skim milk were added to each well and incubated O/N 4˚C in the sealed plates. For PACE4-altCT ELISA, 100µL of 15 µg/mL of rabbit biotinylated anti-PACE4-altCT C-terminal end antibody in PBST containing 1% BSA was used. Plates were again washed 4 times with PBST and incubated 1h at room temperature with horse radish peroxidase (HRP) conjugated streptavidin (ThermoFisher, 1/5000) in PBST. Following 4 others washes with PBST, 100µL of room temperature equilibrated TMB (1-step Ultra TMB ELISA Substrate solution, ThermoFisher) were added to each well and incubated 10 minutes at room temperature prior to the addition of 20 µL 2N sulfuric acid. OD_{450nm} and OD_{540nm} (reference) were then measured for each well in a SpectraMax 190 plate spectrophotometer (Molecular Devices). All value were blank subtracted and divided by their respective OD_{540nm} and used to draw a 4-parameter standard curve using GraphPad Prism (6.0) from which sample concentrations were interpolated and compensated for their respective initial dilution.

Cell culture, RT-qPCR and Western blot

For transfection, cells were plated in 6-well plate (300,000 cells/well) and transfected with Lipofectamine 3000 (Invitrogen) and DNA plasmid (purified using the QIAgen plasmid purification kit following the manufacturer's guidelines). The pMAX deltaN-ERG plasmid was a gift from Valeri Vasioukhin (Addgene plasmid # 29447). 48h after transfection, cells were then washed with PBS and lysed in RIPA buffer. Cell lines stably expressing Non-target shRNA and PACE4 shRNA are documented in Couture et al. (2012)[2]. Their conditioned media were obtained by culturing 75 % confluent 100 mm petri dishes with serum-free RPMI 1640 for 24h. Media were centrifuged to remove any detached cells or debris and were stored at -80˚C until use. Cells were lysed in RIPA buffer over a 20 min incubation on ice (with frequent vortexing) followed by a 30 min centrifugation at 13.000 x g at 4˚C. For Western blot, method. Beta actin (Cell Signaling Technologies, clone 13E5; dilution 1/20,000) was used as a loading control. ERG antibody (clone A7L1G) was purchased from Cell Signaling Technologies and used at 1/1,000 dilution in 5% BSA in TBST buffer. Obtained immunoblots images were

adjusted as whole membrane for brightness and contrast when needed. Full uncropped blots are displayed in Supplementary Figure 4.

For RT-qPCR, RNA was extracted using Qiagen RNA isolation kit (Qiagen, Valentia, CA, USA). Real-time quantitative PCR reactions were performed as previously described in Panet *et al.* (2017)[3]. Primers used are the same as in Couture et al. 2017. [1]

PACE4 MS transition identification and selection

To determine the best transition to monitor PACE4 concentration in samples, immunoprecipitations (IP) followed by LC-MS/MS analysis were performed on lysate of HEK293 cells (1mg) expressing recombinant PACE4 Couture et al. 2017 with anti-P-domain antibody, subjected to trypsin digestion. [1] Briefly, 15µg of biotinylated antibody were bound on Streptavidin conjugated beads (Ocean NanoTech, Springdale, USA) and incubated with the sample in a final volume of 1mL completed with PBST. Beads were washed four times with PBST, three times with 50 mM ammnonium bicarbone (pH 8.0) and digested at 37˚C overnight with 0.2 µg of trypsin/LysC (Promega) in 50µL of this buffer. Bead supernatant was further used for solid-phase extraction prior to LC-MS/MS analysis.

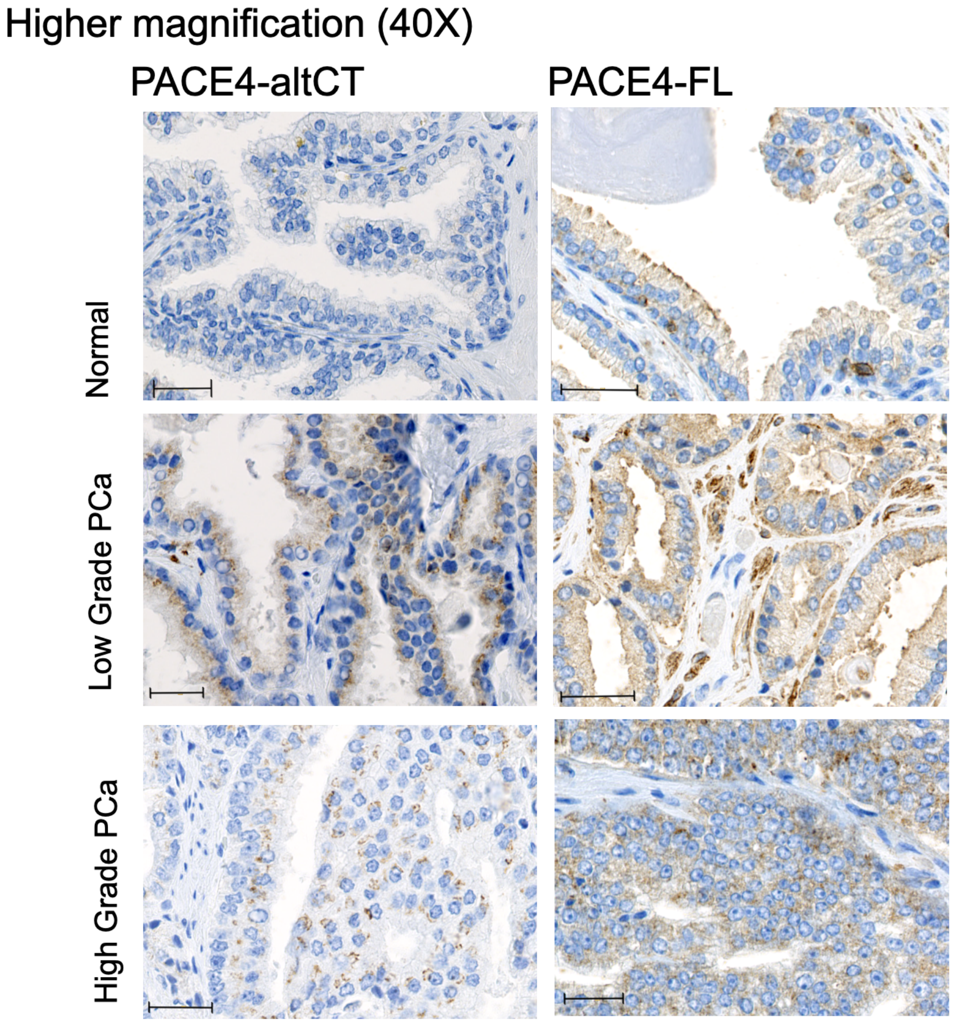

Supplementary Figure 1 – Cellular localization of both PACE4 isoforms

40x magnification of normal prostate, low and high grade PCa encompassing the cellular localization of immunostaining signal. Scale bar represent 25 μm

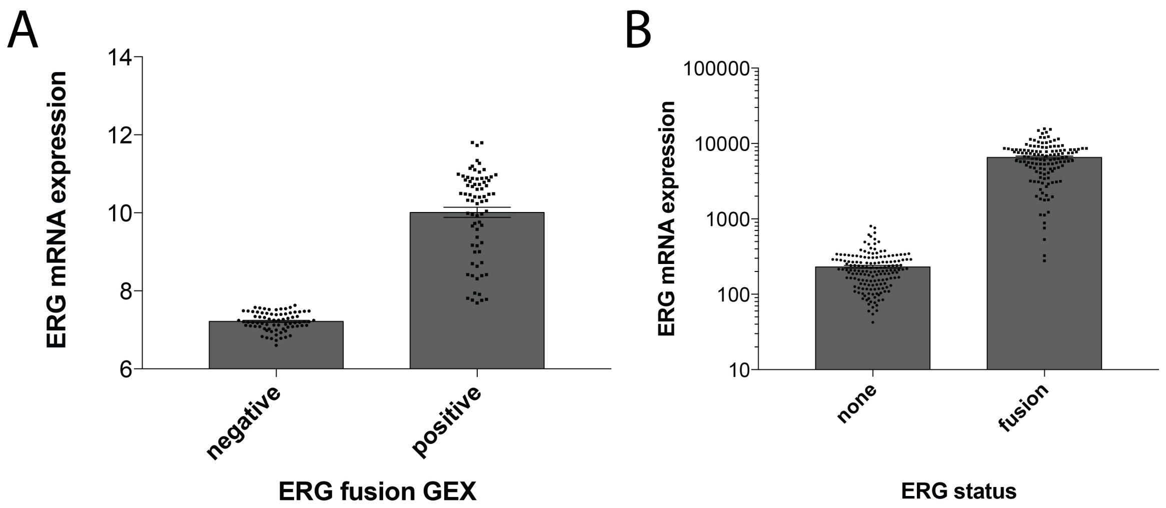

Supplementary Figure 2 – ERG expression following TMPRSS2-ERG gene fusion

A – Histogram view of ERG mRNA expression (log2 whole transcript mRNA expression values using Affymetrix Human Exon 1.0 ST arrays) according to ERG fusion GEX status in the MSKCC, Cancer Cell 2010 dataset and (B) ERG fusion status in the TCGA, Cell 2015 datasets (where mRNA expression is based on RSEM RNA Seq V2 log2 values), both retrieved using cBioportal tool.

A

Amino acid sequence	Number of residu	Conf. (%)	Modification/cleavage	Parent ion (m/z)	Charge (z)
AEGQWTLEIQDLPSQVR	17	99,00		985,50	2
GDLQIYLVSPSGTK	14	99,00		739,38	2
GFGLVDAEALVVEAK	15	99,00	cleaved Y-G@N-term	759,41	2
GPFEGKPIPNPLLGLDSTR	19	99,00		670,03	3
MLELSAPELEPPK	13	99,00		727,38	2
NHPDLAPNYDSYASYDVNGNDYDPSPR	27	99,00		1019,45	3
NVVVTILDDGIER	13	99,00		721,89	2
RGDLQIYLVSPSGTK	15	99,00	missed R-G@1	817,44	2
VVVTILDDGIER	12	99,00	cleaved N-V@N-term	664,87	2
WTAVPSQHMCVAASDK	16	99,00	Carbamidomethyl(C)@10	894,41	2

B

Transitions	Parent ion (Q1)	Fragment ion (Q3)
MLELSAPELEPPK	727.38	809.5
	727.38	880.47
NVVVTILDDGIER	721.89	1031.5
	721.89	1130.5

C

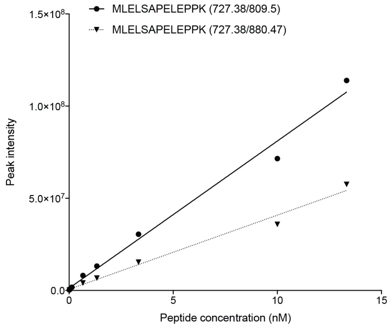

D

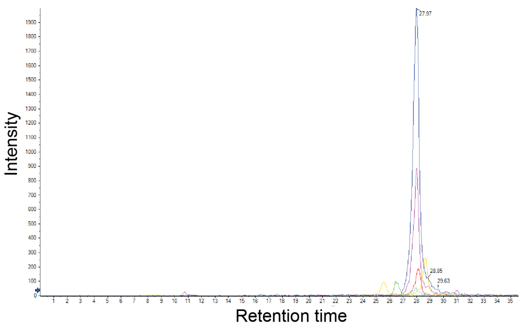

Supplementary Figure 3 – PACE4 tryptic peptide transitions selection for PACE4 titration

A – List of all PACE4 derived tryptic peptides identified with at least 99% confidence.

B – Best transitions selected for multiple reaction monitoring (MRM)

C – Typical standard curves of two distinct MRM transitions used to determine PACE4 concentration in purified recombinant preparation.

D – Obtained single ion chromatogram with increasing concentrations of peptide for the MLELSAPELEPPK peptide monitored using the 727.38/809.5 transition.

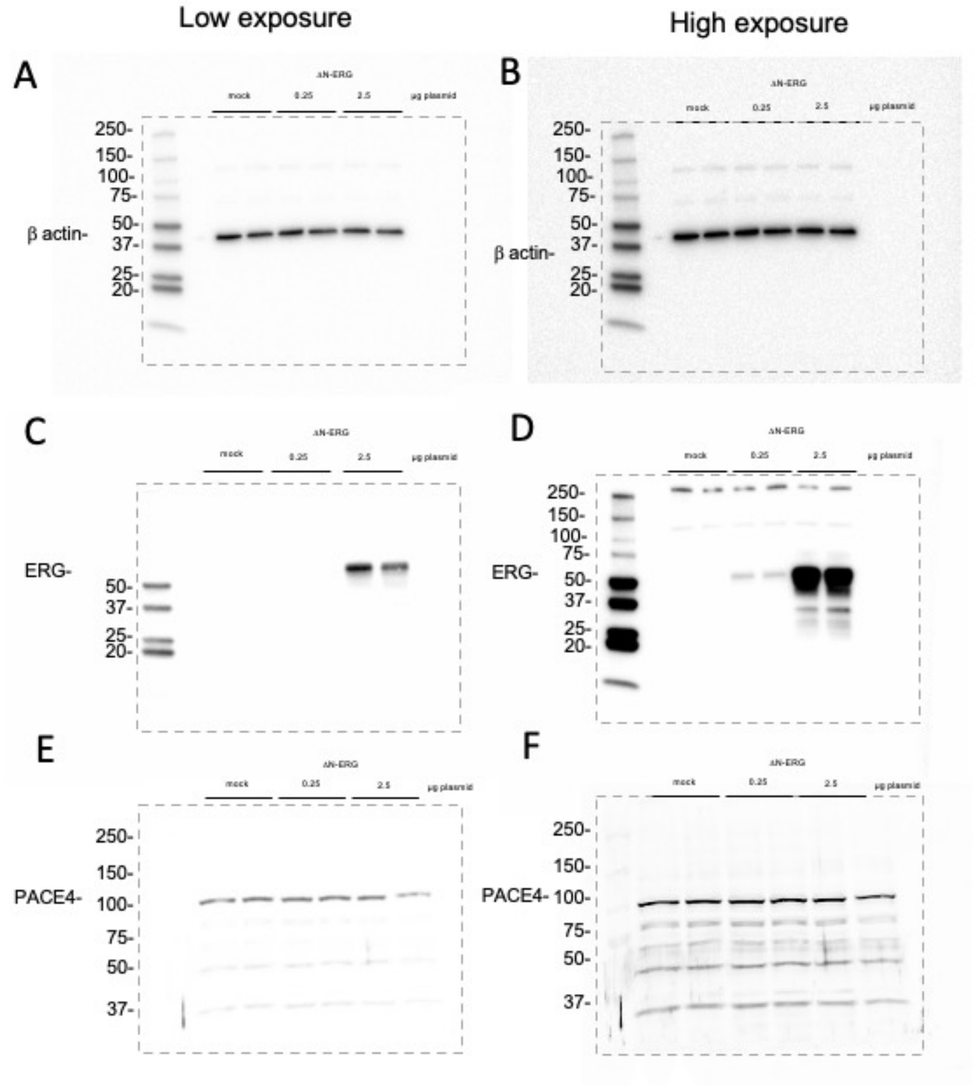

Supplementary Figure 4 – Uncropped blots from Figure 1A

(A-B) Single channel, uncropped blots, displayed with two distinct exposure, for beta-actin, (C-D) for ERG and (E-F) for PACE4. The membrane were not cut prior to hybridization. The tick-lines are there to help see where the full membrane extend.

Supplementary references

1 Couture F, Sabbagh R, Kwiatkowska A, Desjardins R, Guay S-P, Bouchard L *et al.* PACE4 Undergoes an Oncogenic Alternative Splicing Switch in Cancer. *Cancer Research* 2017; **77**: 6863–6879.

2 Couture F, D'Anjou F, Desjardins R, Boudreau F, Day R. Role of Proprotein Convertases in Prostate Cancer Progression. *Neoplasia* 2012; **14**: 1032–IN6.

3 Panet F, Couture F, Kwiatkowska A, Desjardins R, Guérin B, Day R. PACE4 is an important driver of ZR-75-1 estrogen receptor-positive breast cancer proliferation and tumor progression. *Eur J Cell Biol* 2017. doi:10.1016/j.ejcb.2017.03.006.
